# Immersion-plated palladium nanoparticles onto meso-porous silicon layer as novel SERS substrate for sensitive detection of imidacloprid pesticide

**DOI:** 10.1038/s41598-021-88326-0

**Published:** 2021-04-28

**Authors:** A. M. Al-Syadi, M. Faisal, Farid A. Harraz, Mohammed Jalalah, Mabkhoot Alsaiari

**Affiliations:** 1 Promising Centre for Sensors and Electronic Devices (PCSED), Advanced Materials and Nano-Research Centre, Najran University, P.O. Box: 1988, Najran, 11001 Saudi Arabia; 2 Department of Physics, Faculty of Science and Arts, Najran University, Najran, Saudi Arabia; 3 Department of Electrical Engineering, Faculty of Engineering, Najran University, Najran, Saudi Arabia; 4 Nanomaterials and Nanotechnology Department, Central Metallurgical Research and Development Institute (CMRDI), P.O. 87, Helwan, Cairo, 11421 Egypt; 5 Department of Chemistry, Faculty of Science and Arts, Najran University, Najran, Saudi Arabia; 6 Department of Chemistry, Faculty of Science and Arts At Sharurah, Najran University, Najran, Saudi Arabia; 7 Physics Department, Faculty of Education, Ibb University, Ibb, Yemen

**Keywords:** Nanoscale materials, Techniques and instrumentation

## Abstract

Herein, we demonstrate the effectiveness of surface-enhanced Raman scattering (SERS) to detect trace concentration of potentially harmful imidacloprid pesticide. To achieve this ultimate objective, a rapid and highly effective methodology for the fabrication of active and stable porous silicon (PSi) plated palladium nanoparticles (PdNPs) SERS substrates by an electrochemical anodization and immersion plating routes was applied. The PSi layers were fabricated by the electrochemical anodization of a silicon wafer in ethanoic fluoride solution, followed by uniformly deposition of PdNPs via a simple immersion plating technique. The structural features and morphology of fabricated frameworks of PSi-Pd NPs have been investigated by field emission scanning electron microscopy (FE-SEM) with energy dispersive X-ray (EDX), X-ray diffraction (XRD), X-ray photoelectron spectroscopy (XPS) and Fourier transform infrared (FT-IR) spectra. The PSi substrate demonstrates a meso-porous morphology with good distribution, good pore density and average pore sizes around 20 nm. The SERS performance of Si–Pd NPs and PSi–Pd NPs substrates has been examined taking imidacloprid (an insecticide) as a target analyte. The SERS signal of imidacloprid using PSi–Pd NPs substrate exhibited immense enhancement compared to the Si-Pd NPs substrate. The active substrate revealed excellent detectable performance with a concentration as low as 10^–9^ M imidacloprid and an enhancement factor (EF) of 1.2 × 10^5^. This large EF is fundamentally ascribed to the combined effect of the electromagnetic improvement and charge transfer mechanisms. Additionally, no aging effect was observed for the present substrates kept in air for two weeks. Striking enhancement in Raman spectral signals obtained with the current PSi–Pd NPs substrates can provide a simple and smooth platform towards the sensitive detection of various target analytes.

## Introduction

One of the essential benefits of Raman spectroscopy (RS) in comparison to other vibrational spectroscopic approaches is its capability to extract fingerprint information from complex compounds. Nevertheless, the low signal conversion efficiency, low scattering cross section and high fluorescence interference of the analyte led to low Raman signal and weak response during the detection. The surface-enhanced Raman scattering (SERS) technique depending on the plasmonic properties of the surface of the transition metals and noble metals (ordinarily Co, Ni, Ag, Cu, Au and Pd nanoparticles) has been extensively exploited towards amplification of Raman signals^1–6^. Therefore, applying SERS, the highly concerning issue of the comparatively weak sensitivity of RS was lastly overwhelmed, and SERS is widely accepted as a reliable technique to get molecular data from biological and chemical molecules on surfaces as well as in solutions^7^. In SERS, the enhancement of Raman signal can be attributed to two proposed mechanisms: chemical mechanism (CM) and electromagnetic mechanism (EM). The CM is based on chemical effect possessing specified interactions between the metal particles and analyte molecules^1,7–9^, whereas the EM approach is fully dependent on the electromagnetic effect. The significant improvement in electromagnetic field at or nearby the laser-brightened noble metal particle surfaces can be ascribed due to localized surface plasmon (LSPR) excitement (‘‘hotspots” at a surface of metal), generating more strong Raman scattering from molecules nearby or adsorbed onto the particle surface^1,7,8^. Under such a situation, the SERS strength is intensely sensitive to the particle size, composition, arrangement, shape and surface structure^7^. Improvement in Raman signal by surface plasmon resonance was predominantly from metallic substrate, like gold (Au), silver (Ag) or palladium (Pd) nanoparticles. Because of the unsteady movement of solution-based metal nanoparticles, a flat substrate like Au-painted glass substrate could be promising and became appropriate and commercially obtainable SERS substrate. Nevertheless, the features of utilizing metallic nanostructure have considerable recognition in SERS detection with ultra-sensitive responses^4,10–12^. Exploitation of transition metals like Pd for the amplification of SERS signal is considered to be significant approach with promising future prospective of Raman application to improve novel SERS active substrate with high sensitivity^3,13^. On the other hand, much research has been focused on porous silicon (PSi) layers SERS substrates to explore its highly desirable characteristic features like open porous structures and its massive interior surface area^1,14^. PSi layer that is often fabricated via an electrochemical etching route^14–17^ is highly recommended and found to be promising candidate due to ease in fabrication, several attainable pore sizes and morphologies, large surface area and controllable surface modulation and reactivity^16^. Several Au, Ag or Pd nanostructures were decorated onto PSi layers through several methods, such as chemical deposition, thermal decomposition or via immersion/dipping plating^1,7,16,18^. Among deposition techniques, the immersion plating is advantageous due to its simple synthesis methodology and self-induced deposition without requirement for energy supply or vacuum equipment^16^. In a recent report, the synthesis of SiO_2_ capped Ag NPs on Si as SERS active substrates for in-situ cellular DNA detection was demonstrated^19^. Yue et al.^20^ reported a sputtering technique to fabricate Ag onto PSi as an active SERS substrate for the sensitive detection of R6G. Further details on recent development of SERS-active substrates based on metal-coated PSi are well documented^21^.

During the last few years, disastrous effect of imidacloprid on honey bee increases the concern of scientific community and regulation authorities and rated pesticide imidacloprid as a threat to commercial honeybee colonies. In 2018, studies on environmental and human health danger valuations for Thiamethoxam, neonicotinoids, Clothianidin and Imidacloprid by EFSA confirmed that use of neonicotinoid (insecticide) on outside crops is highly dangerous to honeybees and overland bees^22^. Consequently, EU governments completely banned active neonicotinoid including imidacloprid on outside crops due to their lethal effect on pollinators. Therefore, early detection of these toxic neonicotinoid is highly recommended. Present concern urgently required very simple-straight forward methodology to enable fast, reliable and precise recognition or detection of these harmful moieties. Imidacloprid (IDP), [1–6(Chloro-3-pyridylmethyl)-N-nitroimidazolidin-2-ylideneamine], is commonly used pesticide discovered in the firstly 1990s. It is widely used as a pesticide against whiteflies, leafhoppers, plant hoppers, thrips and aphids^23–25^. It closely looks like neurotransmitter acetylcholine and capable to merge with acetylcholine receptors (nAChRs: a signal receiver extant in neuron) target and damaged signal transition for the insect leading to death or paralysis^24,25^. Detection of imidacloprid usually requires expensive approaches like high performance liquid chromatography (HPLC) or HPLC-mass methods because of their volatility or weakness thermal stability^26–28^. These techniques are time consuming and require lots of efforts and technical skills. Thus, it is hard to detect diverse polarity multi-class pesticides, such as imidacloprid, with one unpretentious extraction method and instrument^26^. Fortunately, SERS was found to be a highly potent and benchmark technique to overcome and fulfil the criteria for the present development.

In this work, we report a highly vital, cost-effective, simple and reliable approach for the fabrication of Pd nanoparticles (Pd NPs) decorated PSi layers as an active and stable SERS substrate. The PSi layers with mesoporous structure were fabricated by a simple electrochemical anodization approach. Decoration of Pd NPs onto PSi layer was attained by a simple immersion plating technique with uniform deposition of nanoparticles onto the porous substrates. In addition, Pd NPs were deposited on flat Si wafer surface to examine the influence of Pd NPs onto non-porous Si substrate and to understand the role of porous layer in SERS enhancement. SERS investigation has been carried out using Si-Pd NPs and PSi-Pd NPs for the detection of imidacloprid. This highly recommended facile and straightforward fabrication methodology could provide a smooth platform for the development and designing of PSi-Pd NPs SERS substrate, having prosperous future in detection of various other target molecules.

## Experimental section

PSi layers were synthesized by the electrochemical anodization using *p*-type Si wafer < 100 > orientation with a resistivity of 0.0045–0.006 Ω cm and thickness of 450 ± 25 μm, fabrication procedure is well documented in literatures^29,30^. Briefly, the Si wafers were cut into specific size (1.2 × 1.2 cm squares), rinsed in distilled water and sonicated in acetone for 20 min, followed by dipping in 5wt% aq. hydrofluoric acid (HF) to remove the native oxides. The electrochemical etching was done by placing the Si wafers in a Teflon cell utilizing a copper plate as a back electrode and a small O-ring to close the Si wafer to the cell, revealing an area of nearly 0.8 cm^2^ to the solution (28wt% of aq. HF in absolute ethanol). A platinum (Pt) wire was dipped in the solution as a counter electrode. A constant current density of 6.25 mA/cm^2^ was turned on for 10 min and no supplementary thermal or chemical treatments was done after the galvanostatic anodization. The cell and as-synthesized specimen were subsequently rinsed by ethanol and dried. Pd NPs deposition onto non-porous Si and PSi surfaces was achieved utilizing immersion plating methodology in an aqueous solution of 1 × 10^−3^ M Pd (CH_3_COO)_2_ at room temperature for different immersion times mainly (10, 20,30, 40, 50, 60, 70 and 80 s). The prepared samples were carefully washed by distilled water and dried naturally.

The field emission scanning electron microscopy, FE-SEM, (FESEM; JSM-7600F-JEOL) was employed to observe the pore size and to study the morphology of PSi-Pd NPs. The X-ray diffraction (XRD) patterns have been done on PANalytical X’ port diffractometer using Cu Kα_1/2_, λ_kα1_ = 154.060 pm, λ_kα2_ = 154.439 pm radiation to investigate the surface structure and crystalline nature of deposited samples. The X-ray photoelectron spectroscopy (XPS) measurements were carried out by utilizing VG Escalab200R electron spectrometer equipped with Mg Kα X-ray source (*hν* = 1253.6 eV) and a hemispherical electron analyzer working at fixed transmission energy (20 eV). The Fourier transform infrared spectroscopy (FT-IR) spectra were recorded by means of a spectrometer (100 Perkin Elmer-FT-IR) for Si, PSi and PSi-Pd NPs.

The respective substrates were incubated in 20 μL of different molar concentrations of imidacloprid as a target analyte for 60 min and dried in air. Raman spectrometer (a Perkin Elmer Raman Station 400) was employed to estimate the SERS response. Concerning the 300-mW laser with 785 nm, 60% of its power was utilized with 20 s a combination time and 10 μm as a laser spot size.

## Results and discussions

### Physicochemical characterization of Pd NPs coated-PSi substrates

**Figure 1:**
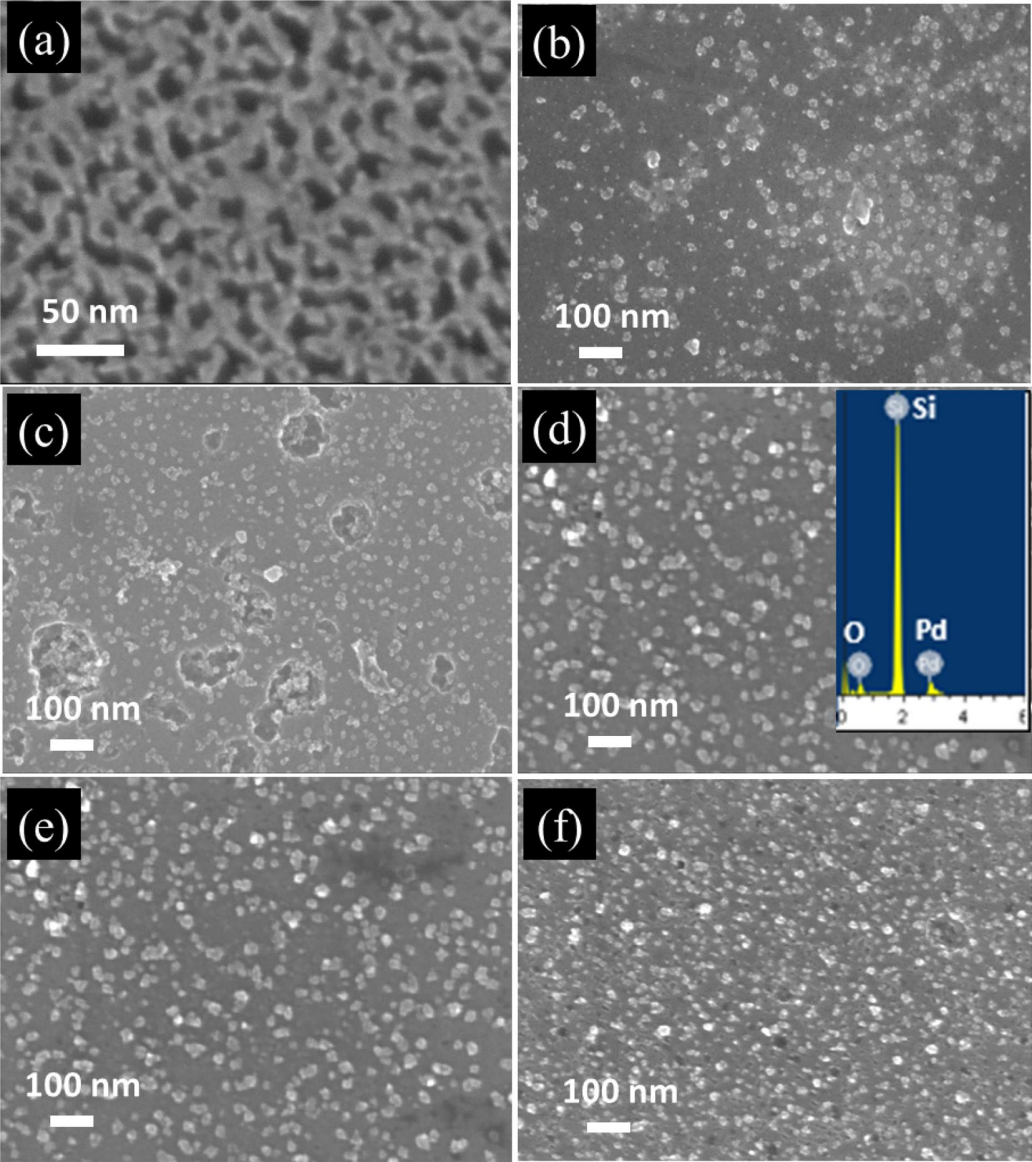
(**a**) FE-SEM image of as-synthesized PSi; FE-SEM images of Pd NPs obtained by immersion process of PSi in Pd(CH_3_COO)_2_ solution at different immersion times: (**b**) 10 s, (**c**) 20 s, (**d**) 40 s, (**e**) 60 s and (**f**) 80 s; (*inset* **d**) EDX spectra for the sample obtained after 40 s immersion plating.

The FE-SEM micrograph of as-synthesized PSi is displayed in Fig. 1a. This micrograph exhibits a meso-porous morphology with good distribution, good pore density and average pore sizes in the range of 20 nm. The meso-porous structure would afford a tremendously great number of nucleation spots for the growth of the metallic nanoparticles. The immersion plating of Pd proceeds directly after exposition of PSi layers to the Pd^+^ ions solution. The pores are obviously disconnected with a short pore-to-pore space. This small pore size as well as predictable large surface area of meso-PSi play a crucial role for absorption and penetration ability through the sensing procedure^16,31^. The FE-SEM images of PSi-Pd NPs at constant Pd^+^ ions concentration for various immersion plating times (10, 20, 40, 60 and 80 s) are illustrated in Fig. 1b-f respectively. It could be observed that the surface of PSi coated with Pd NPs through the immersion plating process resulted in the deposition of Pd NPs onto the PSi layer with grain size ranging nearly between 15 and 30 nm. From these images, it has been noted that with increasing the immersion time from 10 to 80 s, the density of Pd NPs deposit increases. At the immersion time 80 s (Fig. 1f), the pores on PSi layer was tremendously covered by the Pd NPs. The energy dispersive X-ray (EDX) spectrum of PSi-Pd NPs substrate immersion-plated for 40 s is displayed as an inset in Fig. 1d. As revealed, Pd, Si and O elements are predominantly detected. This EDX analysis confirms the existence of Pd NPs on the PSi layer. The appearance of a small spectral line of O in the EDX analysis is likely owing to the aging effect of the PSi layer where some of the Si–H bond could be transformed to more stable bond such as Si–O^3^.

**Figure 2:**
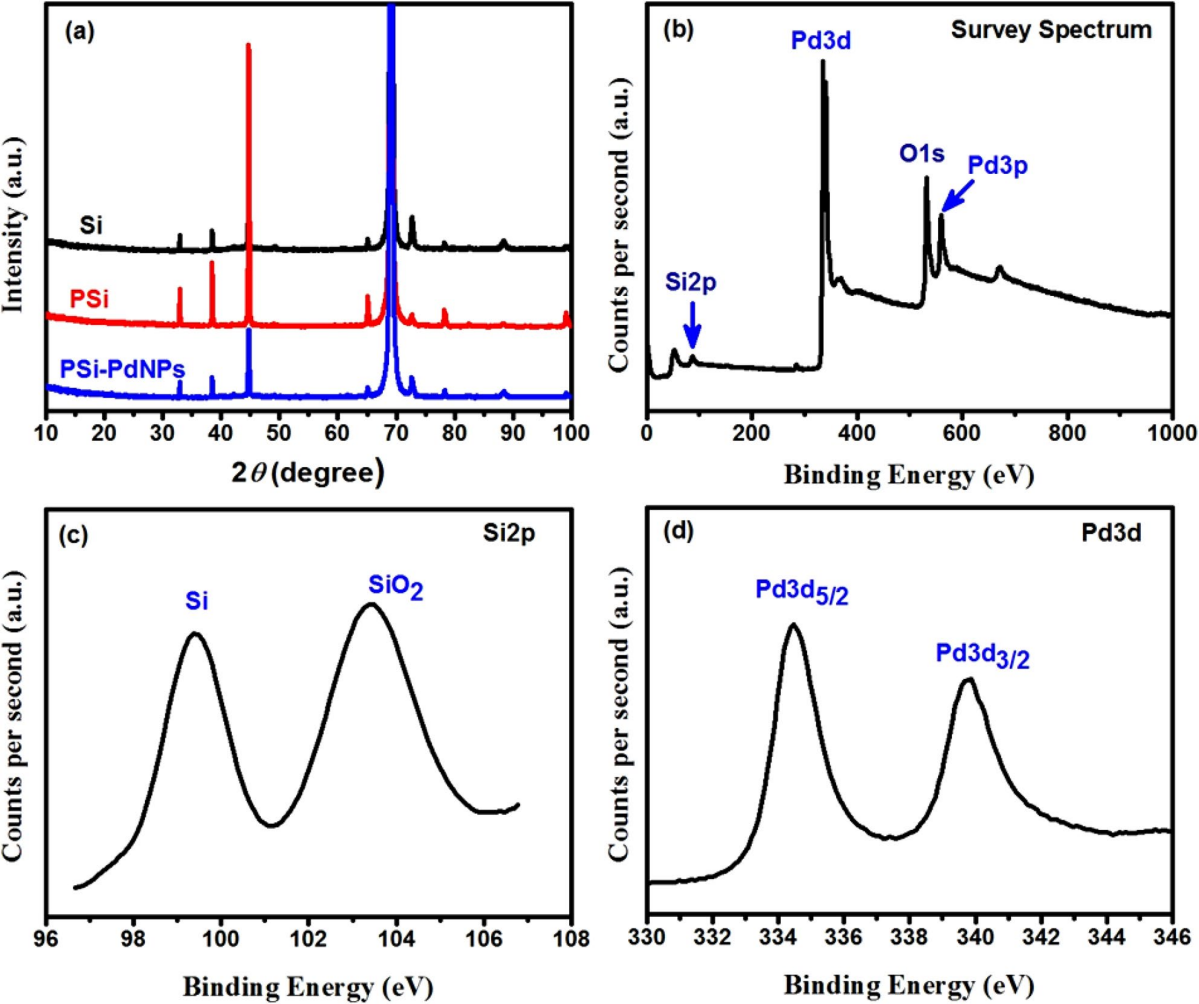
(**a**) The XRD patterns of Si, PSi and PSi-Pd NPs substrates, (**b**) survey spectrum (wide-scan) and narrow-high resolution scan XPS spectra of the as-synthesized PSi-Pd NPs, the high resolution scan spectra were measured for the: (**c**) Si2p and(**d**) Pd3d.

The XRD patterns obtained for starting Si wafer, PSi and PSi-Pd NPs substrates are presented in Fig. 2a. All XRD patterns displayed diffraction peaks at 32.8°, 38.5°, 44.7°, 65.1°, 69.2°, 72.6°, 78.3° and 88.4°. These diffraction peaks are ascribed to the Si crystalline structure^32,33^, except for two low-intensity peaks at 65.1°and 72.6° that may be related to SiO_2_ [PDF#00–001-0438] due to the possible oxidation during samples transfer. The highest peak shown at 2*θ* = 69.2° in each substrates is attributed to Si structure and the presence of this peak in all substrates suggests that the cubic structure of the crystallized Si is preserved even after the pore creation into the Si wafer^34^. After depositing Pd NPs onto the PSi layer, the XRD pattern of PSi-Pd NPs also exhibits the same XRD diffraction bands for Si and PSi substrates, no peaks associated to the Pd NPs have been recognized, suggesting the low concentration of Pd NPs into PSi-Pd NPs substrate or the small diffraction bands in comparison to the Si-related peaks.

The XPS measurements were carried out to get information on the electronic state and the composition of the surface region of PSi-Pd NPs sample. As shown in Fig. 2b, the XPS survey spectrum indicates evident peaks of Si (2p), Pd (3p), Pd (3d) and O (1 s), which emphasize the existence of Pd NPs deposited onto the PSi layer. The appearance of O (1 s) peak at 529.5 eV is associated with the oxide oxygen^35^. The XPS spectrum of the Si (2p) in Fig. 2c reveals double peaks with binding energies at 103.7 eV and 99.6 eV, attributing to SiO_2_ and (Si^0^), respectively^15,36^. Si oxidation is a synchronous reaction taking place with the Pd^2+^ ion reduction and is commonly disclosed during the immersion coating of noble metals into the PSi surface^15,36^. The final, fabricated substrates are consequently a partly oxidized PSi layer plated with Pd NPs. The redox reactions of the plating procedure could be accordingly characterized via the following formula:

$$ 2{\text{Si}} - {\text{H}}_{{\left( {{\text{surface}}} \right)}} + {\text{H}}_{2} {\text{O}} = {\text{Si}} - {\text{O}} - {\text{Si}}_{{\left( {{\text{surface}}} \right)}} + 2{\text{H}}_{{\left( {{\text{aq}}} \right)}}^{ + } + 2{\text{e}}^{ - } \quad \left( {{\text{oxidation}}} \right) $$

$$ {\text{Pd}}^{2 + } + 2{\text{e}}^{ - } = {\text{Pd}}_{{\left( {\text{s}} \right)}} \quad \left( {{\text{reduction}}} \right) $$

In Fig. 2d, the XPS spectral lines of the Pd (3d) illustrates double peaks with binding energies at 334.5 eV and 339.8 eV, attributing to Pd3d_5/2_ and Pd3d_3/2_, respectively. Compared with the standard spectrum of metallic Pd, the binding energies of Pd^0^ rise slightly (from 334.5 to 335.9 eV and from 339.8 to 341.2 eV), which may result from the substrate-nanoparticle interphase polarization effects^37^. These results are in consistent with the anticipated values for Pd^0^ and confirmed that Pd^2+^ ion has been perfectly reduced to metallic Pd^0^ utilizing the immersion plating method^38^.

**Figure 3:**
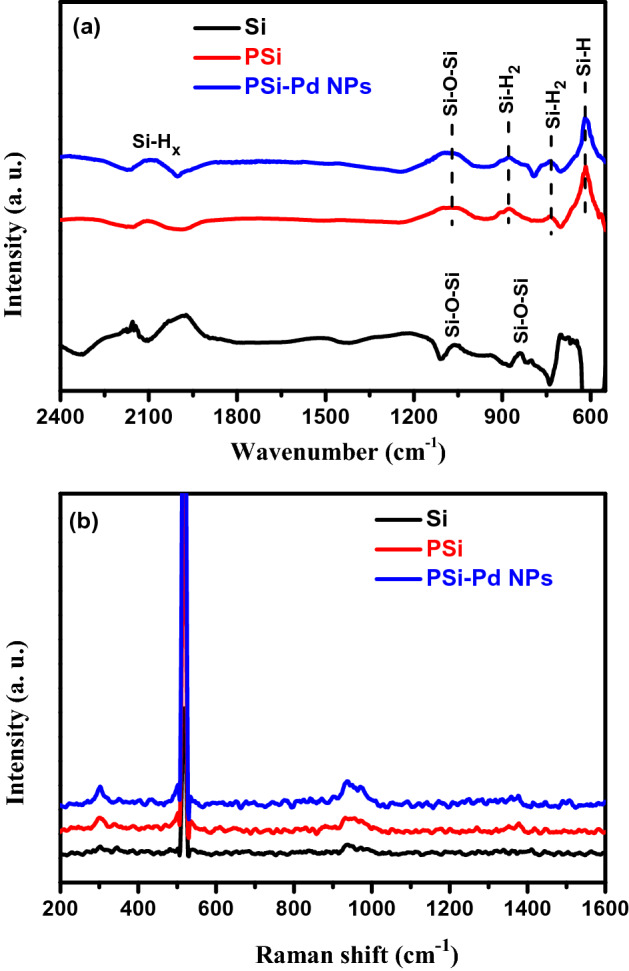
(**a**) FT-IR spectra and (**b**) Raman spectra of Si, PSi before and after Pd plating.

The FT-IR spectra of Si, as-prepared PSi and PSi-Pd NPs substrates in the range 550–2400 cm^−1^ are displayed in Fig. 3a. In the case of Si substrate, the spectrum displays Si–O–Si bond at 1070 cm^−1^ and 838 cm^−1^^17,39^, which could be due to the possible oxidation during sample transfer^16^. In the case of the PSi-Pd NPs and PSi substrates, one can conclude that the internal surface of the as-synthesized PSi is composed of Si hydrides species, SiH_x_. The obtained spectra show the Si–H bond at 625 cm^−1^, the Si–H_2_ bond at 732 cm^−1^ and 875 cm^−1^ and Si–H_*x*_ bonds at 2060–2150 cm^˗1^, which in good agreement with previous reports^3,16,17,40^. These Si hydride-correlated bands represent the characteristic features of a typical PSi single layers^17^. The FT-IR signal of PSi-Pd NP is resemble to that obtained from PSi layer. No peak related to Pd NPs has been observed indicating the low concentration of Pd NPs in PSi-Pd NP combination. Furthermore, the peak detected at 1070 cm^−1^ is related to the Si–O–Si, and likely generated due to the oxidation of Si as a counter reaction during the displacement reaction of Pd or due to the aging effect of the PSi layer in ambient atmosphere.

Raman spectra of Si, PSi and PSi-Pd NPs substrates are displayed in Fig. 3b. The spectra of PSi and PSi-Pd NPs substrates are fairly similar to that of crystallite Si with one strong intense band at ~ 520 cm^−1^ originating from the optical-phonon scattering at the center of the Brillouin zone of Si^41^. As displayed in the spectra of PSi and PSi-Pd NPs substrates, the band detected at ~ 945 cm^−1^ may be correlated to transverse optical phonons scattering^1^. The spectrum shows a small peak at ~ 295 cm^−1^, which could be allocated to crystalline Si^42^. Moreover, the nonappearance of the band correlated to *a*-Si which demonstrates ordinarily at 480 cm^−1^ emphasizes the crystallinity of all samples.

### SERS evaluation of Pd NPs coated-PSi active substrates

**Figure 4:**
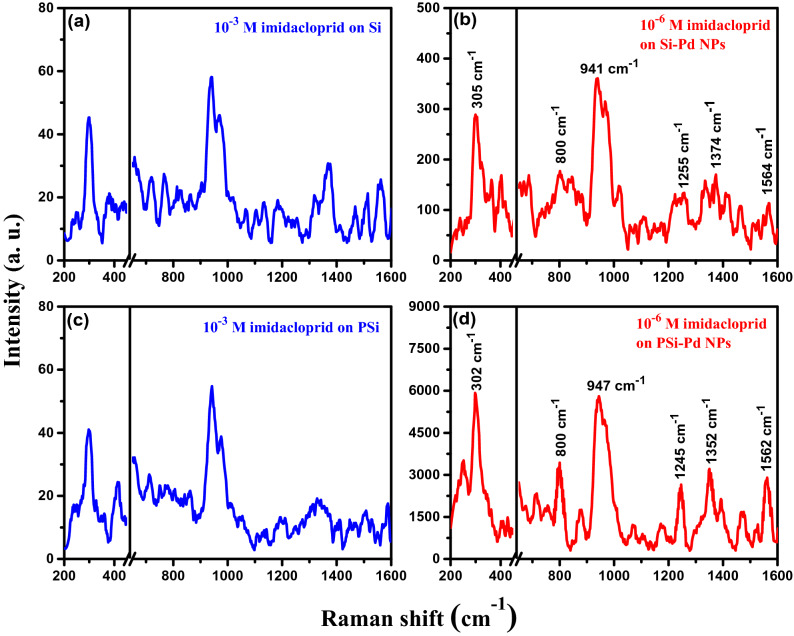
Raman spectra of the: (**a**) Si, (**b**) Si-Pd NPs, (**c**) PSi, and (**d**) PSi-PdNPs substrates after incubation in specific concentration of imidacloprid.

The SERS behavior governed by the morphology of porous structures is highly dependent on the density of hotspot regions, where there is amplification in electromagnetic field due to energy transfer between imidacloprid molecules and Pd NPs. To confirm the capability of PSi–Pd NPs as an active SERS substrate, a SERS investigation has been conducted utilizing bare PSi (without Pd NPs) as a control substrate, whereas Si wafers with and without Pd NPs were also employed as the reference substrates. Figure 4 displays the Raman spectra obtained from imidacloprid on Si wafer with and without Pd NPs (Fig. 4a,b) as well as PSi layer with and without Pd NPs (Fig. 4c,d). It is pertinent to mention here that bare Si and PSi substrates were examined at high concentration i.e. 10^–3^ M of imidacloprid whereas the Si and PSi with Pd NPs were studied at lower concentration i.e. 10^–6^ M of imidacloprid. Si-Pd NPs and PSi–Pd NPs substrates exhibited an extremely high intense peaks compared to bare substrates i.e. without metallic Pd NPs. Additionally, PSi–Pd NPs substrate showed expressively intense peaks (i.e. reflecting the excellent performance as a SERS substrate). Furthermore, investigation of insecticide imidacloprid (10^–6^ M) with Pd NPs coated Si and PSi utilizing the immersion plating technique yielded much more intense imidacloprid fingerprint peaks (Fig. 4b,d). Imidacloprid allocated peaks for the Si–Pd NPs substrates were observed at 305, 800, 941, 1255, 1374 and 1564 cm^−1^, whereas for PSi–Pd NPs substrates, the peaks were found at 302, 800, 947, 1245, 1352 and 1562 cm^−1^ (as demonstrated in Fig. 4b,d, respectively) demonstrating a slight shift in peak position. Nevertheless, the spectra of imidacloprid for both SERS and normal Raman are in good agreement with previous available reports^22,23,43^. The assignments of Raman bands of imidacloprid are listed in Table 1. As can be seen, the peak intensities produced on both SERS substrates (Si-Pd NPs and PSi–Pd NPs) reflect an outstanding advancement which is ascribed to the electromagnetic and chemical improvement occurring due to the charge transfer mechanism between the absorbed imidacloprid and Pd nanocrystallites onto the Si-Pd NPs and PSi–Pd NPs substrates. The highly improved electromagnetic features are notably obtained due to the hotspots generated at the connections between the substrate surface components or among the Pd NPs collected in the Si and PSi substrates^7,44,45^. The above results clearly confirm that the presence of Pd NPs on Si wafer or PSi layer is the key constituent for SERS improvement. Among the two substrates, the PSi induced substrate was found to be more effective as compared to Si wafer, although both SERS substrates were initially coated with Pd NPs. The porous surface (possessing high surface area) and densely packed nanoparticles in PSi–Pd NPs substrate supported the efficient generation of highly recommended hotspots compared with Si–Pd NPs substrate resulted in enhancement and improvement of SERS signals^1,10^. In addition to this, the high intense signal utilizing PSi–Pd NPs originates due to porous structure, size and density of the Pd NPs on the PSi layer which could help to create greater number of hotspots at the inter-particle connections^1,10^. Therefore, this PSi-Pd NPs substrate was further utilized for the rest of SERS investigation and measurements.

**Table 1:** Assignments of Raman bands of imidacloprid in SERS and normal Raman conditions.

Raman	SERS Si-Pd NPs	SERS PSi-Pd NPs	Assignment
316	305	302	N–C–N bond out-of-plane vibration
817	800	800	C–H in-plane rocking mode
989	941	947	C–N stretching
1273	1255	1245	N–N stretching
1363	1374	1352	C–N stretching
1555	1564	1562	C–H in-plane bending vibration

**Figure 5:**
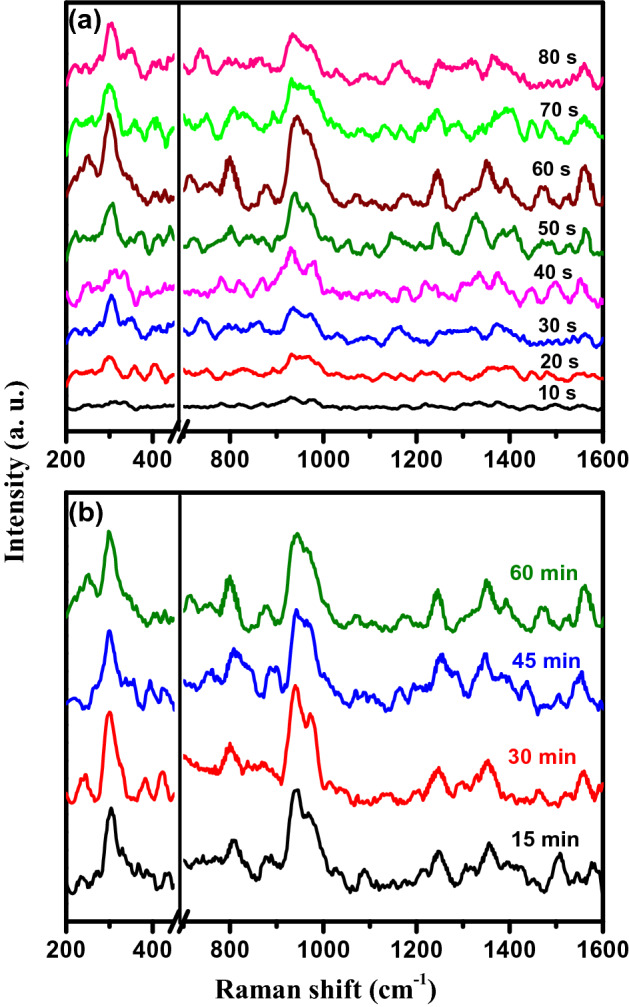
SERS spectra of 10^−6^ M imidacloprid collected from PSi-Pd NPs substrates: (**a**) fabricated at different Pd dipping times, (**b**) using different incubation times.

Figure 5a exhibits the SERS spectra obtained from a fixed concentration of imidacloprid (10^–6^ M) on PSi-Pd NPs substrates prepared at different immersion times (from10 to 80 s) in Pd ion solution for a constant incubation time (60 min) of imidacloprid. As illustrated in the figure, the peak intensities of SERS spectra improved greatly with the variation of Pd NPs distribution and concentration. The highest SERS spectrum is associated to the substrate prepared utilizing 60 s immersion time. The Raman peaks obtained from this substrate appear at ⁓ 302, 800, 947, 1245, 1352 and 1562 cm^−1^, which can be assigned to Raman bands of imidacloprid, as listed in Table 1. Therefore, this PSi-Pd NPs substrate was utilized thereafter in the rest of SERS measurements and evaluation. Furthermore, the variation in immersion time i.e. shorter or longer times than 60 s, the substrates showed reduced performance which may be due to the excitation wavelength that might take place out or inside the resonance plasmonic band of the Pd NPs in PSi substrate^46^. Substrate with 60 s immersion time displays higher density of Pd NPs with smaller inter-particle separation distance and subsequently generating dynamic inter-particle coupling effects and ultimately enhancing the spectral intensity of SERS^46^. In case of shorter immersion time than 60 s, the amount of Pd deposited onto the PSi layer is relatively low. Therefore, this substrate shows less active or negligible response towards the SERS spectrum enhancement. At the immersion time 60 s, the inter-nanocrystals Pd dimension reaches to an enhanced state that could interfere the electromagnetic field to create hotspots at the nanocrystals connections, therefore, created hotspots intermediated SERS signal magnification of the adsorbed imidacloprid molecules. In case of longer immersion time than 60 s, the corresponding SERS signal intensity of imidacloprid molecules was considerably low. This could be due to covering the pores of PSi layer by increasing the Pd NPs deposit, which led to a decrease in surface area of PSi and suppress the role of PSi in enhancement the SERS spectra. On the other hand, the dense packing of Pd NPs could lead to diminish the interparticle dimension due to the formation of a continuous dense layer of Pd NPs, as a bulk metallic Pd. Consequently, the improvement of SERS signal was not achieved due to minimized formation of hotspots^46^. Figure 5b displays SERS spectra obtained from a fixed concentration of imidacloprid (10^–6^ M) on PSi-Pd NPs substrates for different incubation times (15, 30, 45 and 60 min) of imidacloprid. It can be observed that the SERS spectra obtained at various imidacloprid incubation times are almost similar to each other, highly suggesting the uniform adsorption of imidacloprid onto the PSi-Pd NPs substrates. Therefore, it could be confirmed that the parameter of various incubation times has no obvious effect on SERS spectra and therefore the incubation time less than 60 min also seems suitable for the worthy detection of imidacloprid under the current experimental conditions.

**Figure 6:**
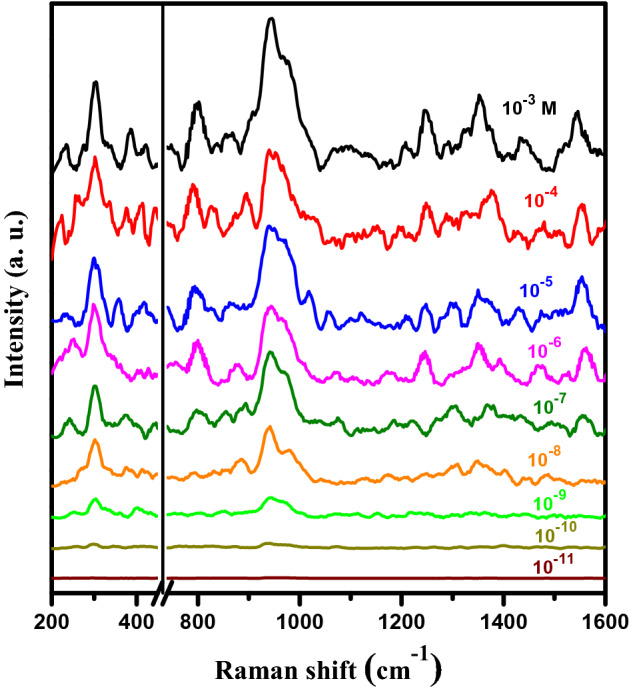
SERS spectra collected at different concentrations of imidacloprid onto PSi-Pd NPs substrates.

Figure 6 illustrates the SERS spectra of PSi-Pd NPs active substrate with various molar concentrations of imidacloprid (from 10^−3^ to 10^−11^ M). Obvious peaks were detected at imidacloprid lower concentrations 10^−9^ M. Nevertheless, when target concentration is reduced to either 10^−10^ M or 10^−11^ M, the major SERS spectral features of imidacloprid were hardly detected. This is probably due to the reduction of the effective imidacloprid molecules at the hotspot regions, which would lead to reduce the SERS intensity^1,41^. Thus, the lowermost detectable “ensured” concentration of imidacloprid is 10^−9^ M.

**Figure 7:**
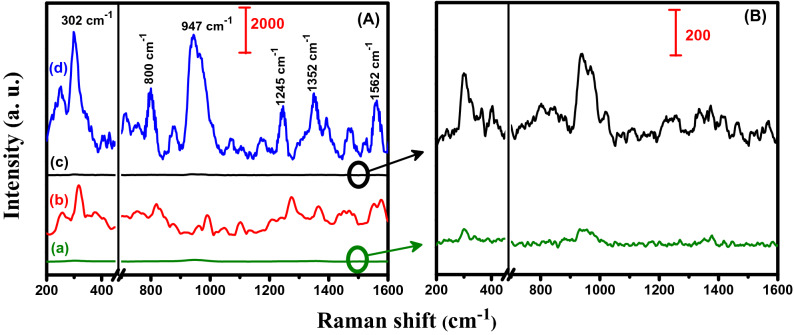
In (**A**), (a) Raman spectrum of 10^−6^ M imidacloprid collected on bare PSi as a control, (b) Normal Raman spectrum of imidacloprid powder, (c) SERS spectrum of imidacloprid (10^−6^ M) obtained from Si-Pd NPs substrate and (d) SERS spectrum of imidacloprid (10^−6^ M) obtained from PSi-Pd NPs active substrate. For clarity, in graph (**B**), the spectrum of bare PSi substrate (a) and for Si-Pd NPs substrate (c) are displayed in a different scale.

Figure 7 illustrates the Si-Pd NPs and PSi-Pd NPs SERS spectra obtained from 10^−6^ M imidacloprid, collected with the Raman signal of imidacloprid bulk powder as well as a control experiment utilizing a bare, unmodified PSi substrate. As demonstrated in spectrum (a) in Fig. 7A, there was almost no SERS improvement observed for unmodified PSi substrate, a magnified spectrum is also shown in Fig. 7B. Raman signals of imidacloprid bulk powder, as demonstrated in spectrum (b) of Fig. 7A, display distinguished peaks correlated to the imidacloprid spectral features. The SERS spectrum of imidacloprid utilizing Si-Pd NPs is shown as spectrum (c) in Fig. 7A. From this spectrum, it was observed that there was no clear peaks appeared due to the small peak intensities for this spectrum compared with the high response of PSi-Pd NPs substrate (see above Fig. 4b,d). The spectrum of (c) is re-drawn in a different, magnified scale in Fig. 7B and revealed characteristic peaks related to imidacloprid but in small intensities. In case of SERS signals obtained from PSi-Pd NPs substrate, as demonstrated in Fig. 7d of collected graph (A), all peak intensities are meaningfully improved, confirming a significant improvement of Raman spectra using the active surface of PSi-Pd NPs substrate.

The process of SERS is pertinent to the factor of analytical enhancement. Therefore, the ratio between the intensity of the SERS and Raman signal for target molecules refers to the analytical enhancement factor (EF). The EF was estimated for the current substrate by utilizing the following equation^1,47,48^.

$$ EF = \frac{{I_{SERS} \times N_{bulk} }}{{I_{bulk} \times N_{surf} }} $$

where *I*_SERS_ is the intensity of SERS of imidacloprid absorbed on the PSi-Pd NPs layer, while *I*_bulk_ is the intensity of the Raman signal for imidacloprid bulk powder. *N*_bulk_ is the average number of molecules participating to the normal Raman spectrum, *N*_surf_ is the average number of target molecules under laser spot focus due to SERS measurement. The area used for absorbing imidacloprid molecules is almost 0.4 nm^2^ for the vertical orientation^1,49^ with a long-axis length of nearby 1.4 nm^1,50^. The *N*_bulk_, beneath the area of laser spot (∼10 μm in diameter and 2 μm in penetrating depth), was estimated to be 2.8 × 10^11^ molecules; {*N*_bulk_ = π × 25 × 2 μm^3^/(0.4 × 1.4 nm^3^)}. To evaluate *N*_surf_, 20 μL of a 10^−6^ M imidacloprid solution was permeated onto 0.8 cm^2^ of PSi-Pd NPs. Assuming that all imidacloprid molecules are absorbed onto the PSi-Pd NPs substrate, *N*_surf_ is calculated to be 1.2 × 10^7^ molecules; [*N*_surf_ = 20 μL × 10^−6^ mol L^−1^ × π × 25 μm^2^ × 6.02 × 10^23^/0.8 cm^2^]. By taking or relying the upper intensity peak at 947 cm^−1^, the *I*_SERS_/*I*_bulk_ ratio is calculated to be 5.3 (Fig. 7). Therefore, the EF value obtained from the present PSi-Pd NPs substrate for imidacloprid was estimated to be 1.2 × 10^5^. It is worthy to notice that the evaluated SERS EFs are commonly dependent on the excitation wavelengths as well as the type of target molecule under investigation. It has been revealed that this obtained EF locates in the range of reported values for metallic nanocrystals on solid substrates, which are in the range of (10^4^–10^7^)^1^. The enhancement of the present SERS measurements is likely correlated to the existence of “hotspots” on the PSi-PdN Ps SERS substrate. The hotspots could yield very highly enhancement of local field and they have been exposed by examination nanocrystallized metal employing powerful microscopes^44,45^. The existence of Pd NPs distributed on the PSi layer yields to the generation of “hotspots”, which in turn might yield to spectrum magnification. Relying on the morphology of substrate, it could be supposed that “hotspots” are produced at two diverse levels. “Hotspots” because of Pd NPs, with small Pd inter-particle distance, in the pores and “Hotspots” due to connected pathway between two or more pores. Consequently, the SERS signals are formed on a large detection confocal volume and thus it is assumed that both types of “hotspots” take part to the improvement of SERS spectrum^7^. Generally, the enhancement of Raman signal during SERS measurement can be attributed to two proposed mechanisms: electromagnetic mechanism (EM) and chemical mechanism (CM). The significant improvement in SERS spectrum comes from EM, which is generally considered to be independent of the target molecule. In contrast, the CM depends intensely on the specific molecule and the local environment of the metal surface because it results from the overlap between the wave functions of the metal nanoparticles (Pd NPS) and the target molecule (imidacloprid). This overlap outcomes in a renormalization of the molecular orbitals as well as the introduction of new mixed charge-transfer (CT) states. Both of these effects will contribute to the CM enhancement of the Raman spectra and can be categorized as the non-resonant chemical mechanism and a resonant charge-transfer chemical mechanism, respectively^51,52^. It is worthy to mention here that the EFs calculated using either Si wafer or PSi substrates (without Pd deposition) were found to locate around unity, however the EF for Si coated with Pd NPs was estimated to be 7.5 × 10^3^, compared to the above high EF value of 1.2 × 10^5^ calculated for the active Pd NPs coated PSi substrate. This result demonstrates the decisive role of porous structure as well as the presence of Pd NPs for the current SERS improvement. The SERS enhancement behavior along with the LOD of the current PSi-Pd NPs substrate for the detection of imidacloprid is compared with previously reported SERS substrates as listed in Table 2^3,23,26,43,53–56^.

**Table 2:** Comparison of SERS performance for various substrates toward the detection of different analytes (where n/a = not applicable).

SERS substrate	Analyte	Limit of detection (LOD)	Enhancement factor (EF)	References
PSi-Pd NPs	Imidacloprid	10^−9^ M	1.2 × 10^5^	This work
Ag-nanoflower (Ag-NF)	Imidacloprid	3.9 × 10^−10^ M	n/a	^23^
Au NPs/plant surfaces	Imidacloprid	2 × 10^−9^ M	n/a	^26^
Ag/PVDF substrates	Imidacloprid	1.6 × 10^−9^ M	n/a	^43^
Au NPs/Polymethacrylate	Imidacloprid	3.9 × 10^−5^ M	n/a	^53^
Pd NPs/PSi	TNT	10^−7^ M	2.5 × 10^6^	^3^
Pd film	Benzenethiol	n/a	1.8 × 10^3^	^54^
Ag–Pd alloy nanostructures	Rhodamine 6G	10^−9^ M	2.6 × 10^8^	^55^
Pd nanoboxes	4-Mercaptopryidine	n/a	1.3 × 10^4^	^56^

**Figure 8:**
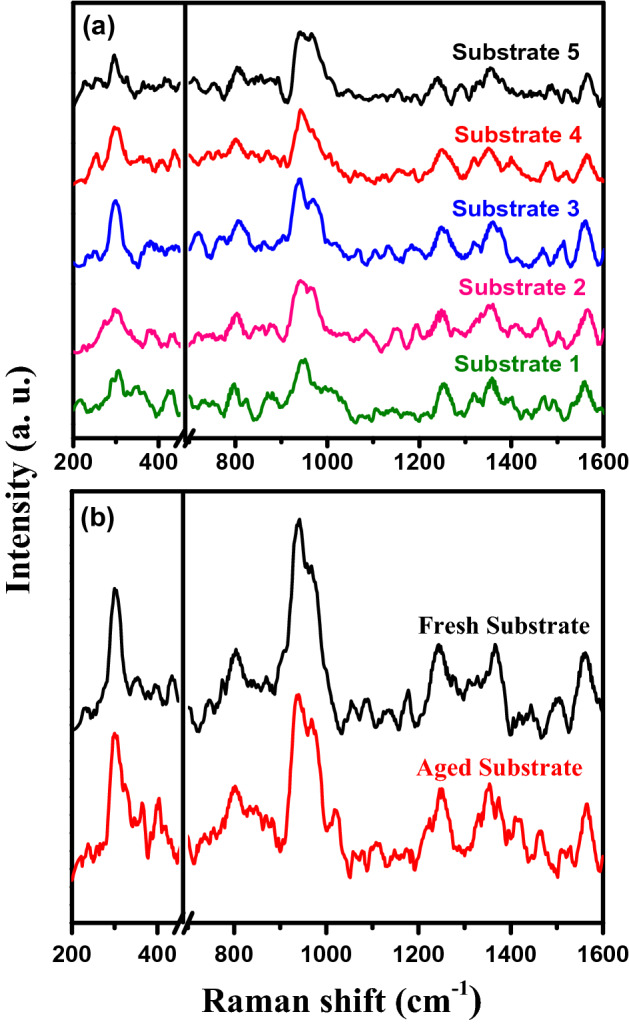
(**a**) Reproducible SERS spectra of 10^−6^ M imidacloprid collected from various PSi-Pd NPs substrates prepared under identical conditions. (**b**) SERS spectra of 10^−6^ M imidacloprid obtained from fresh and aged PSi-Pd NPs substrates. The substrate that has been kept in air for two weeks still displays high and comparable sensitivity to the fresh substrate.

**Figure 9:**
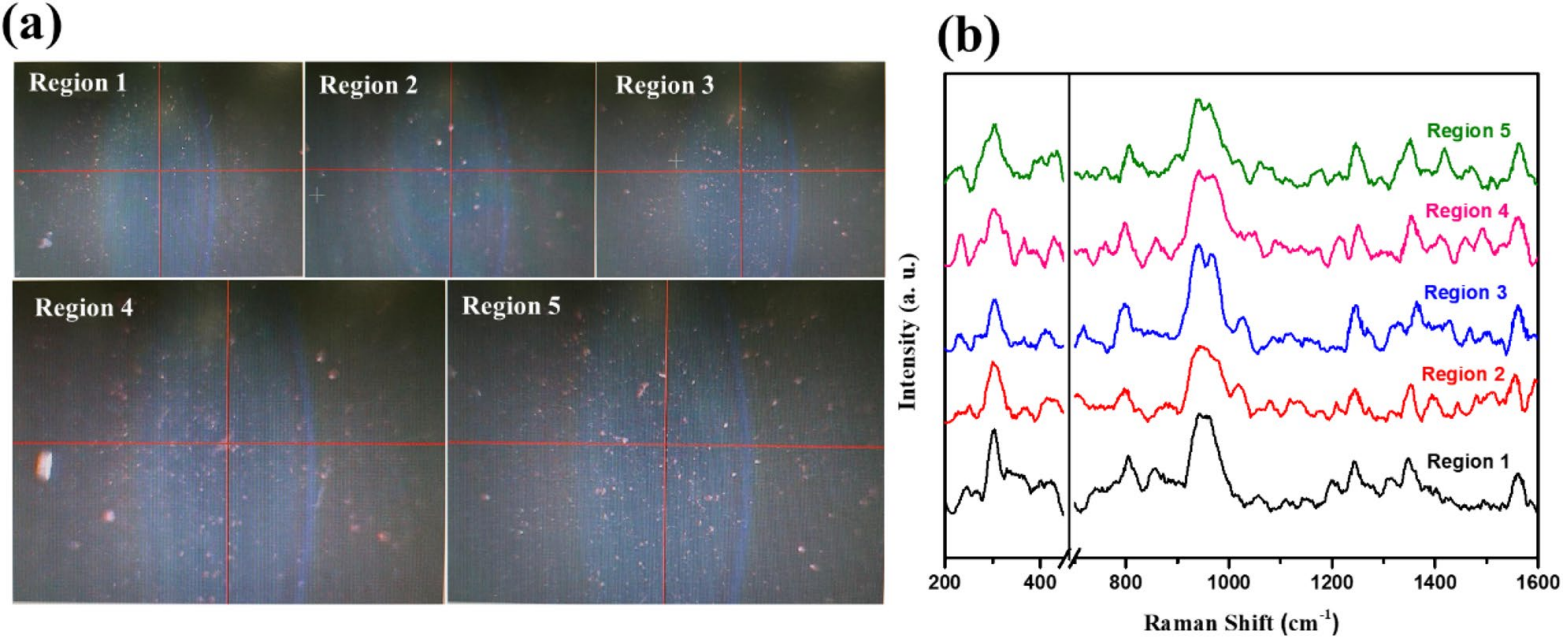
(**a**) Optical images (dimensions: 1.7 × 2.8 mm) of PSi-Pd NPs substrate at different regions. (**b**) The corresponding SERS spectra of 10^−6^ M imidacloprid collected from PSi-Pd NPs substrate at five different regions.

The SERS spectra were further collected using five different substrates fabricated under identical conditions to evaluate the reproducibility, as shown in Fig. 8a. We noticed that the spectra have well-distinguished peaks, identically resemble to each other. The aging effect was also tested for a substrate kept in air for two weeks. The SERS signal of aged substrate using 10^−6^ M imidacloprid exhibited no substantial spectral variation compared to the fresh substrate, as displayed in Fig. 8b. This result indicates the good stability and good reproducibility of the current PSi-Pd NPs SERS active substrate. Furthermore, the SERS signals which measured at five different regions of the active substrates are quite similar, indicating good uniformity of Pd coating and porous matrix, as revealed from Fig. 9.

**Figure 10:**
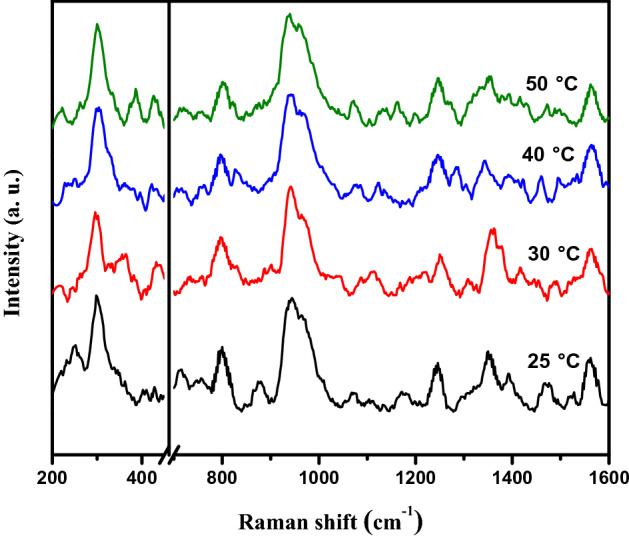
SERS spectra of 10^−6^ M imidacloprid collected from PSi-Pd NPs substrates heated at different temperatures mainly; 25, 30, 40 and 50 ℃ for 15 min.

Figure 10 shows SERS spectra obtained from a constant concentration of imidacloprid (10^–6^ M) on PSi-Pd NPs substrates heated at different temperatures (25, 30, 40 and 50 ℃) for 15 min. As observed, the SERS spectra obtained at different substrate temperatures are quite similar to each other. Therefore, this result proved that the changing temperature from 25 up to 50 ℃ has no obvious effect on SERS spectral response and consequently suggested the operational stability of the substrate within the examined temperature range (25—50 ℃).

## Conclusions

In this study, a facile, inexpensive and rapid detection approach for imidacloprid pesticide was developed using SERS active substrate based on Pd NPs coated meso-PSi layers. The electrochemical anodization of silicon wafer was applied to produce meso-porous structures of PSi layers, whereas the Pd NPs were effectively deposited via a simple immersion plating method. The same strategy was also applied to fabricate Si–Pd NPs SERS substrate utilizing flat Si as a substrate. Highly uniform and sensitive SERS signals have been obtained from imidacloprid molecules adsorbed onto PSi–Pd NPs active substrates, which displayed a remarkable SERS improvement compared with either flat Si-Pd NPs or uncoated PSi substrates. The detection limit of imidacloprid molecules by the current PSi–Pd NPs substrate was 10^−9^ M with an EF of 1.2 × 10^5^ and excellent stability and reproducibility. This improved SERS performance is likely related to the uniform distribution and arrangement of Pd NPs onto PSi substrate, which brought greater number of electromagnetic hotspots at the inter-particle connections. The current fabrication approach is promising to be adopted for further synthesis of highly sensitive SERS substrates with other metallic nanostructures for sensitive detection of various chemical and biomolecules.
